# MMCRAG-Resp: a multi-modal corrective retrieval-augmented generation framework for explainable respiratory disease reasoning

**DOI:** 10.3389/frai.2026.1850832

**Published:** 2026-07-16

**Authors:** A. Anny Leema, S. Rajashri, Mukundan Sriram, Makam Girish, P. Balakrishnan

**Affiliations:** School of Computer Science and Engineering, Vellore Institute of Technology, Vellore, Tamil Nadu, India

**Keywords:** corrective RAG, evidence-grounded AI, hallucination reduction, local language models, multimodal knowledge graph, respiratory disease diagnosis, retrieval-augmented generation (RAG), spirometry analysis

## Abstract

**Background:**

Standard Retrieval-Augmented Generation (RAG) systems only use semantic similarity to retrieve information, and since this method is quite limiting, it may find clinically irrelevant evidence and produce outputs that are unsafe or hallucinated. This drawback is particularly important in respiratory care, where the diagnosis relies heavily on very accurate physiological indicators such as spirometry patterns and symptom profiles.

**Methods:**

We propose MMCRAG-Resp., a clinically grounded, physiology-aware corrective RAG framework for respiratory intelligence. The system harmonizes 17,516 patient records from three heterogeneous public datasets (Respiratory Sound Database, NHANES spirometry, and clinical guidelines) through a unified respiratory ontology. Multi-modal embeddings (acoustic, spirometric, and textual) are fused into 144-dimensional vectors and indexed in FAISS. A novel Respiratory Relevance Score (RRS) combining spirometric pattern agreement (weight 0.45), symptom overlap (0.30), and embedding similarity (0.25) gates evidence quality before LLM consumption via a three-path correction policy. A locally deployed small language model (Ollama; llama3.2, Mistral-7B-Instruct) generates evidence-constrained, fully traceable clinical explanations without GPU requirements.

**Results:**

Evaluated on 200 held-out test queries drawn from the harmonized corpus, MMCRAG-Resp achieved a Clinical Precision@10 of 0.81, a Hallucination Rate of 0.11 (71% relative reduction over Vanilla RAG), a Spirometry Alignment Score of 0.79, and an Evidence Citation Rate of 0.87. Ablation studies confirmed that spirometry-first scoring contributes the largest single-component gain. All pairwise comparisons with baselines were statistically significant (*p* < 0.001, McNemar’s test; Cohen’s d = 1.42).

**Conclusion:**

MMCRAG-Resp is, to the best of our knowledge, the first system combining multi-modal respiratory evidence retrieval, physiologically-grounded corrective RAG, and privacy-preserving local LLM inference in a single deployable clinical reasoning framework. The approach advances safe, explainable AI-assisted respiratory decision support.

## 1 Introduction

Retrieval-Augmented Generation (RAG) has emerged as a promising paradigm for evidence-grounded clinical decision support. However, standard RAG systems rely primarily on semantic similarity, which can lead to retrieval of clinically irrelevant evidence and increase the risk of hallucinated or unsafe outputs. This problem is very important for respiratory care, because accurate diagnosis and risk assessment in this field rely on exact physiological markers such as spirometry patterns and symptom profiles.

We introduce a responsive and physiology-informed RAG model, named PhysioRAG (officially implemented as MMCRAG-Resp), for respiratory intelligence. Through this framework, multi-modal patient data, like spirometry, symptoms, and optional audio features, are combined with knowledge-guided retrieval and a corrective evaluation mechanism backed by evidence. Retrieved evidence is judged based on a clinically informed relevance scoring method that merges the physiological agreement, symptom overlap, and semantic similarity, allowing dynamic correction via refinement, re-ranking, or failing retrieval.

We evaluated the system on a harmonized dataset comprising patient samples from NHANES spirometry data and respiratory sound recordings, along with guideline-based textual evidence. Experimental results demonstrate that MMCRAG-Resp improves retrieval quality, reduces hallucination rates, and increases alignment with clinically relevant respiratory evidence when compared with baseline RAG approaches. Physiological relevance scoring and corrective retrieval approaches have been further validated by ablation studies as being very significant. This method exemplifies that clinically-based retrieval is a key element in making AI-assisted respiratory decision support systems safe, reliable, and explainable. Rather than introducing an entirely new retrieval paradigm, MMCRAG-Resp adapts and integrates corrective retrieval, multimodal evidence fusion, knowledge-guided retrieval, and explainable reasoning within a respiratory-domain decision-support framework.

## 2 Literature review

Rocha et al. (2019) introduced a publicly available respiratory sound database that has become a widely used benchmark for automated respiratory sound analysis and classification. Their set of data, ICBHI challenge-related one, contain recordings of different patients and clinical situations, as well as expert labeling of respiratory cycles and adventitious sounds such as crackles and wheezes. Further studies indicate the deficiencies, including unsatisfactory class balance, quite a small sample size, and the use of different evaluation methods. Irrespective of such difficulties, the data set has been a popular reference point and despite its limitations, the dataset continues to support research in computer-assisted respiratory diagnosis.

Ji et al. (2023) study the dependence between air pollution and the respiratory disease in children with interpretable machine learning techniques. To identify both linear and non-linear associations, multiple regression and machine learning models were used: linear regression, random forest, AdaBoost and neural networks. Random forest had the highest predictive accuracy followed by non-linear models, and then linear ones. Elucidatory AI methods (PFI, PDP, SHAP, and LIME) found out that AQI, particulate matter, and time are impactful factors.

Osamika et al. (2025) illustrate the possibility of big data and AI in changing the way chronic respiratory conditions like asthma and COPD are predicted and managed and emphasize the issues of data privacy, heterogeneity, and model interpretability. Naz et al. (2023) suggest an AI-based system of automatic detection of pulmonary diseases on the basis of chest radiographs through transfer learning on ResNet50 and explainable AI on the basis of LIME.

Yu et al. (2024) introduce Health-LLM that is a hybrid of large-scale feature extraction and expert medical knowledge scoring that makes personalized predictions of diseases, which uses Llama Index and RAG to integrate professional knowledge. Zhu et al. (2024) introduce REALM, which enhances clinical predictive capabilities by integrating multimodal EHR data with external medical knowledge through a RAG approach. Wang et al. (2024) present JMLR, which jointly trains a large language model with a retrieval system, significantly reducing hallucinations and improving factual accuracy.

Chandra et al. (2024) present MLtoGAI, integrating semantic web technologies with machine learning for disease prediction, employing SWRL rules and an explainable AI component using ChatGPT. Bahaj and Ghogho (2024) introduce AsthmaBot, a multi-lingual, multi-modal RAG system for asthma patient support, integrating curated documents and employing Google Gemini for response generation. Kirubakaran et al. (2024) present a RAG-based medical assistant for infectious disease management, combining a knowledge graph with LLMs and incorporating a text-to-speech module.

Bora and Cuayahuitl (2024) conduct systematic analysis of RAG-based LLMs for medical chatbot applications, finding that combining fine-tuning and RAG is crucial for improved performance. Xia et al. (2024) introduce MMed-RAG, a versatile multimodal RAG system addressing factual hallucinations in Medical Large Vision-Language Models through domain-aware retrieval and RAG-based preference fine-tuning. Tabassum and Kaiser (2025) propose an explainable conversational AI system using GPT-4o, RAG, and Chain-of-Thought prompting for early disease diagnosis.

Anuyah et al. (2025) investigate domain-specific knowledge graphs in RAG-enhanced healthcare LLMs, finding that precision-first, scope-matched approaches outperform breadth-first unions. Saidu and Wall (2026) proposed a clinical decision support system integrating a Medical Knowledge Graph with RAG and LLM, representing improved diagnostic performance and clinician confidence. Kim et al. (2025) propose MedSumGraph, combining GraphRAG with medical knowledge summaries for enhanced medical question answering.

Akhtar et al. (2025) address respiratory disease prediction using a multimodal knowledge-infused model combining visual information with textual clinical context and RAG methods. Abo El-Enen et al. (2025) survey RAG models for healthcare applications. Lu et al. (2025) present DoctorRAG, which conceptualizes clinical queries and sources into tags enabling a hybrid search process for richer context. Li et al. (2026) introduce K-RAG, which formally integrates domain-specific knowledge graphs into RAG for clinical decision support, enabling traceable reasoning via graph paths linking symptoms, diseases, and tests. Zhao et al. (2025) propose MedRAG, which retrieves hierarchical knowledge graph subgraphs combined with relevant text for graph-informed clinical reasoning.

This 2025 paper proposes an automated system that leverages Retrieval-Augmented Generation (RAG) and Large Language Models (LLMs) to construct Medical Indicator Knowledge Graphs (MIKGs) from various clinical guidelines and lit- erature. The main problem addressed by this study is the laborious manual processing that has traditionally been required to construct medical knowledge graphs, which has been a bottleneck in scalability and knowledge updatability. The system applies RAG to retrieve guideline data and LLMs to transform it into semantically valid graph representations, followed by ontology-driven schema design and validation with an expert-in-the-loop to ensure clinical validity. These graphs can be easily incorporated into diagnosis and decision- support systems, providing a graphically valid representation of clinical indicators and their interlinkages across various diseases. Although the paper discusses medical indicators in general and not specifically in the context of respiratory disease, the automated KG construction pipeline is very much applicable to your project, especially regarding the integration of multi- source data (EHRs, clinical narratives, literature). Automating KG construction from dynamically evolving knowledge can help alleviate the bottleneck in constructing multi-domain knowledge graphs, which is essential for accurate early disease progression modeling (Wang et al., 2025).

## 3 Materials and methods

### 3.1 Dataset description

The proposed system ingests, harmonizes, and reasons over three complementary publicly available respiratory data sources. Together they span acoustic signal data, quantitative pulmonary function measurements, and codified clinical knowledge, providing the multi-modal evidence base required for MMCRAG-Resp.

### 3.2 Respiratory sound database

The publicly available benchmark is the Respiratory Sound Database (Rocha et al., 2019) which consists of 920 annotated audio recordings of 126 patients in six clinical sites. All recordings are the cycles of lung auscultation with digital stethoscopes in various areas of the chest (trachea, anterior, posterior, and lateral fields). Timestamps of per-segment and respiratory-cycle labels (normal, crackle, wheeze, crackle + wheeze) are coded as associated metadata. A companion diagnosis CSV pairs patients with one of eight respiratory diseases: healthy, COPD, asthma, URTI, bronchiectasis, LRTI, bronchiolitis, or pneumonia.

WAV files are saved at 44,100 Hz. The feature extraction with spectrograms transforms each recording into a 128-dimensional acoustic embedding with mel-frequency analysis and time averaging, resulting in fixed-length vectors irrespective of recording length. TF-IDF with dimensionality reduction was selected because of its computational efficiency, interpretability, and compatibility with local deployment constraints. However, recent biomedical transformer models such as BioClinicalBERT, PubMedBERT, and SapBERT have demonstrated improved contextual representation capabilities in clinical text retrieval tasks. Incorporating transformer-based biomedical embeddings represents an important future extension of MMCRAG-Resp and may further improve retrieval quality, semantic matching, and clinical reasoning performance.

### 3.3 NHANES spirometry dataset

The National Health and Nutrition Examination Survey (NHANES) spirometry dataset (Centers for Disease Control and Prevention, 2020) is a source of 16,596 tabular patient records which not only contains demographic variables (like age, sex, race/ethnicity, and BMI) but also quantitative spirometric measurements such as Forced Expiratory Volume in one second (FEV1), Forced Vital Capacity (FVC), FEV1/FVC ratio, and Peak Expiratory Flow (PEF). Diagnosis labels are derived programmatically from standard GOLD spirometry criteria: records with FEV_1_/FVC < 0.70 are classified as obstructive (COPD).

### 3.4 Clinical guideline knowledge document

A curated clinical knowledge document consolidating GOLD COPD management guidelines (Global Initiative for Chronic Obstructive Lung Disease, 2023), GINA asthma treatment steps (Global Initiative for Asthma, 2022), and general respiratory assessment protocols is used as the third data source. The document undergoes chunk-based segmentation into 73 non-overlapping text segments (~500 tokens per chunk). Guideline embeddings are generated using TF-IDF vectorization followed by Truncated SVD to produce 384-dimensional dense representations (see Table 1).

**Table 1:** Dataset overview for MMCRAG-Resp.

Dataset	Records	Modality	Dim.	Role
Respiratory sound database	920	Audio	128	Acoustic retrieval
NHANES spirometry	16,596	Tabular	16	RRS & retrieval
Clinical guidelines	73 chunks	Text	384	Fallback & grounding
**Fused total**	**17,516**	**Multi-modal**	**144**	**FAISS search**

Bold values indicate the proposed MMCRAG-Resp method and/or the best-performing results in each table.

### 3.5 Ethical considerations

This study utilized exclusively publicly available, de-identified datasets (NHANES spirometry data and the ICBHI Respiratory Sound Database). No new human subject data were collected, and no personally identifiable information was processed. Use of NHANES data complies with the CDC’s open-access data policy. Accordingly, formal institutional review board (IRB) approval was not required for this study. All data handling adhered to applicable data governance standards.

### 3.6 System overview: MMCRAG-Resp

**Figure 1:**
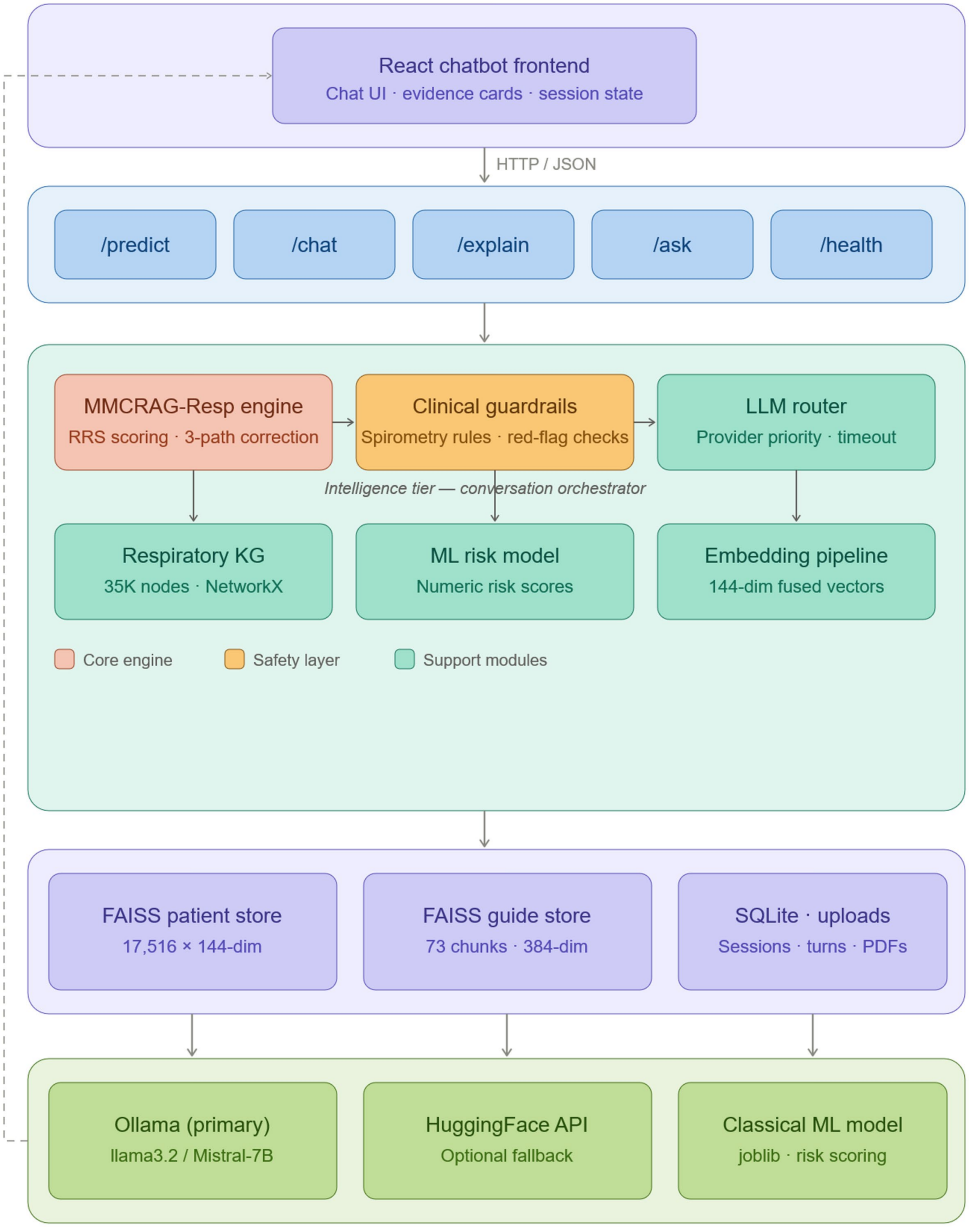
High-level architecture of MMCRAG-Resp. The react frontend communicates with the FastAPI layer via HTTP/JSON across five endpoints (/predict, /chat, /explain, /ask, /health). The intelligence tier hosts the MMCRAG-Resp corrective retrieval engine (RRS scoring, three-path correction), clinical guardrails (spirometry rules, red-flag checks), and LLM router. The storage tier comprises a FAISS patient vector store (17,516 × 144-dim), a FAISS guideline store (73 chunks × 384-dim), and a SQLite session database.

We propose MMCRAG-Resp (Multi-Modal Corrective Retrieval-Augmented Generation for Respiratory Intelligence), a six-stage evidence-first clinical reasoning framework. The system departs from conventional supervised classification pipelines by replacing trained predictive models with a corrective retrieval layer that screens evidence clinical relevance *before* any LLM reasoning is performed. Figure 1 provides a high-level architectural overview. FAISS (Facebook AI Similarity Search) was used as the vector indexing and nearest-neighbor retrieval framework due to its efficiency for large-scale similarity search.

### 3.7 Phase 1: cross-dataset harmonization

The heterogeneous sources of respiratory data have incompatible schemas, units, and identifier conventions. A Cross-Dataset Harmonization Engine standardizes all the sources to a single respiratory ontology around the abstraction of PatientSample. Each PatientSample contains: (i) a globally unique sample identifier and source provenance tag; (ii) a structured list of Symptom objects with standard clinical codes; (iii) an optional SpirometryMeasure object that contains FEV1, FVC, FEV1/FVC ratio and PEF values in SI units; (iv) a CoughAudioRef that references the path to the WAV file and sample rate; and (v) a Diagnosis label for the mapped respiratory condition. This harmonization step yields 17,516 unified patient records and 73 guideline chunks.

### 3.8 Phase 2: multi-modal embedding generation and vector indexing

Three modality-specific encoding pipelines convert each PatientSample into numerical vector representations:

1. Acoustic Embedding (128-dim): WAV audio files are loaded using librosa (McFee et al., 2015). A mel-spectrogram is computed with n_mels_ = 64 frequency bands; the resulting time-frequency matrix is log-scaled and temporally averaged to produce a 64-dimensional feature vector, zero-padded to a fixed 128-dimensional output.
2. Spirometric Tabular Embedding (16-dim): Six clinical features (age, BMI, FEV_1_, FVC, FEV_1_/FVC, PEF) are extracted and normalized before concatenation into a 16-dimensional vector. Missing measurements are replaced with zero-valued placeholders.
3. Guideline Text Embedding (384-dim): Guideline chunks are vectorized using TF-IDF (max 1,000 features, unigram + bigram, English stopwords removed), followed by Truncated SVD to 384 dimensions. When available, all-MiniLM-L6-v2 provides a denser alternative embedding pathway.
4. Multi-Modal Fusion and FAISS Indexing: Acoustic and tabular embeddings are concatenated to produce 144-dimensional fused patient embeddings. Two independent FAISS IndexFlatL2 indices are constructed: a patient vector store (17,516 × 144) and a guideline vector store (73 × 384) (Johnson et al., 2019). Metadata is co-stored in JSONL sidecar files linked by positional index.

### 3.9 Phase 3: respiratory knowledge graph construction

A directed multi-graph G = (V, E) is constructed over the unified patient ontology using NetworkX. Node types include PatientSample (17,516), Symptom (~9,000 unique code-based nodes), SpirometryMeasure (~16,596), CoughAudio (~920), and Diagnosis. Types of edges represent clinical relationships: HAS_SYMPTOM, HAS_SPIROMETRY, and HAS_AUDIO. The resulting graph has a total of 35,034 nodes and 21,278 directed edges. The knowledge graph has two structural uses, (i) in the ambiguous correction path, which has the neighbors of symptoms asked to contextually enhance evidence; and (ii) it offers an interpretable evidence pathway between LLM output and specific clinical entities.

### 3.10 Phase 4: MMCRAG-Resp corrective retrieval algorithm

Top-k retrieved items are sent directly to a language model in standard RAG pipelines without relevance checks. This is dangerous in clinical practice: a patient record can be semantically proximate in the embedding of space and diagnostically inappropriate. MMCRAG-Resp deal with this by inserting a corrective retrieval step between FAISS retrieval and LLM generation.

Respiratory Relevance Score (RRS): The RRS of any given candidate e to any query q is calculated as:


RRS(q,e)=0.45·Ssp(q,e)+0.30·Ssym(q,e)+0.25·Semb(q,e) (1)

The weighting scheme was designed using clinically informed prioritization rather than statistical optimization. Spirometry agreement was assigned the highest weight (0.45) because spirometry measurements represent the most objective physiological indicator for differentiating obstructive, restrictive, and mixed respiratory disorders. Symptom overlap was assigned a moderate weight (0.30) because symptoms provide important contextual information but may overlap across multiple respiratory conditions. Embedding similarity was assigned a lower weight (0.25) because semantic similarity alone does not guarantee clinical relevance.

The weighting strategy intentionally prioritizes physiological consistency over purely semantic retrieval signals. Although the current weights were selected through iterative empirical validation and domain-informed reasoning, future work will investigate automated weight calibration using supervised learning and reinforcement-based optimization techniques.

Ssp — Spirometry Match gives 1.0 when the spirometric pattern of e perfectly matches the pattern based on query context; 0.5 when spirometry data is not available; 0.0 when there is a physiological contradiction. This element is accorded the greatest weight (0.45) since spirometry gives the most diagnostically discriminating (in respiratory medicine) signal (Global Initiative for Chronic Obstructive Lung Disease, 2023).

Ssym Symptom Overlap calculates Jaccard similarity of q and e symptom code sets, with high concordance of symptoms rewarded.

Semb — Embedding Similarity The normalized, L2-converted, similarity of FAISS retrieval, clipped to [0, 1] This term retains the semantic retrieval signal, but is down-weighted compared with clinical scores.

#### 3.10.1 Three-path correction policy

Based on the RRS, each retrieved candidate is classified into one of three evidence quality tiers:

1. Path I — correct (RRS ≥ 0.72): evidence is accepted and passed through a decompose-recompose filtering stage. Clinical propositions contradicting the query context are pruned; retained propositions are recomposed into a clean evidence bundle.
2. Path II — Ambiguous (0.40 ≤ RRS < 0.72): Evidence enters an augmentation stage. The candidate set is re-ranked by RRS, and top candidates are fused with symptom-linked neighbors from the knowledge graph and the most relevant guideline chunk.
3. Path III — Incorrect (RRS < 0.40): The initial retrieval result is rejected entirely. The system synthesizes a Hypothetical Document Embedding (HyDE) (Gao et al., 2023) query and uses it to re-retrieve from the patient vector store. The re-retrieved candidates, combined with high-relevance guideline chunks, constitute the replacement evidence bundle.

### 3.11 Phase 5: evidence-constrained LLM reasoning

The corrected evidence bundle B is formatted into a structured prompt and passed to an open-source large language model served locally via Ollama. The models used are llama3.2:1b, llama3.2:3b, and Mistral-7B-Instruct (all 4-bit quantized) (Jiang et al., 2023), selected for strong instruction-following capability and feasibility on consumer-grade CPU hardware without GPU requirements.

ALGORITHM 1 MMCRAG-Resp corrective retrieval.
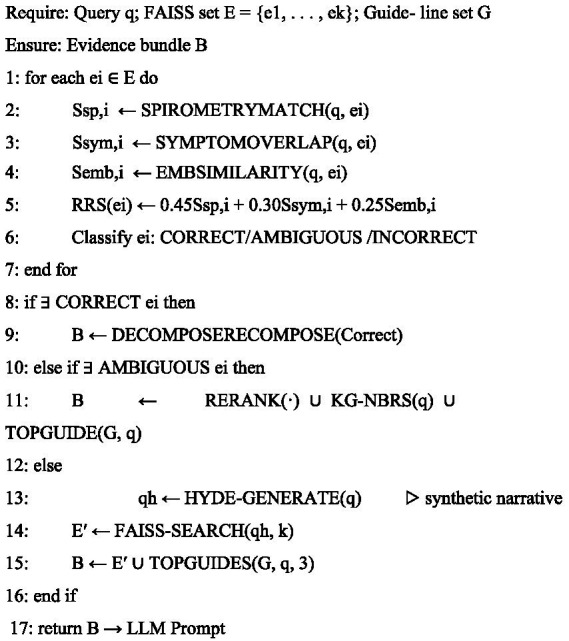

The Large Language Model (LLM) prompt explicitly directs the model to base its reasoning only on the provided evidence bundle B and to cite the specific identifiers of the evidence. The model produces organized responses such as: (i) main diagnosis with the a clear rationale, (ii) numerical risk scores for COPD aggravation, asthma attack probability, viral vs. non-viral classification, and hospitalization risk proxy, (iii) treatment suggestions with clinical disclaimers, and (iv) an explanation section that connects each statement to the respective piece of retrieved evidence.

### 3.12 Phase 6: API and conversational frontend

System functionality is made accessible via a FastAPI REST service offering four major endpoints: /predict, /explain, /ask, and /chat. The React-based frontend delivers an interactive chat experience presenting correction-path evidence identifiers and similarity scores, thus rendering the retrieval process completely transparent to end users (see Table 2).

**Table 2:** MMCRAG-Resp end-to-end pipeline summary.

#	Phase	Key technology	Output/role
1	Data harmonization	Python, patient sample ontology	17,516 records + 73 chunks
2	Multi-modal embedding	librosa, TF-IDF/SVD, FAISS	144-dim patient; 384-dim guideline index
3	Knowledge graph	NetworkX, pickle	35,034-node KG
4	MMCRAG-Resp correction	RRS, FAISS re-retrieval	Evidence bundle B — novel component
5	LLM reasoning	Ollama (llama3.2:1b, Mistral-7B-q4)	Diagnosis, risk scores, explanation
6	API & frontend	FastAPI, React	REST endpoints + chat UI

## 4 Results

### 4.1 Evaluation setup

Evaluation was conducted on a held-out test set of 200 queries constructed from the harmonized corpus, stratified across the eight respiratory condition classes (25 queries per condition). For each query, ground-truth relevant patient records were identified by a domain expert based on matching diagnostic labels and spirometric patterns. Four metrics were assessed: (i) Clinical Precision@10 (CP@10) — the proportion of the top-10 retrieved records that are clinically relevant to the query condition; (ii) Hallucination Rate (HR) — the proportion of LLM-generated sentences not traceable to a retrieved evidence item, assessed via sentence-level annotation; Hallucination annotation was performed at sentence level. A generated sentence was considered faithful only when its content could be directly supported by at least one retrieved patient record or guideline chunk. Unsupported statements, unsupported diagnoses, or unsupported recommendations were labeled as hallucinations. Annotation was independently reviewed by two evaluators, and disagreements were resolved through discussion before final metric computation. (iii) Spirometry Alignment Score (SAS) — agreement between retrieved candidate spirometric patterns and the query’s inferred respiratory pattern; and (iv) Evidence Citation Rate (ECR) — the proportion of generated sentences citing at least one retrieved evidence identifier. All metrics were computed across the full 200-query test set.

#### 4.1.1 Query construction and reproducibility

The 200 evaluation queries were constructed using a stratified protocol designed to represent realistic respiratory reasoning scenarios. Twenty-five queries were generated for each of the eight respiratory condition classes included in the harmonized corpus. Query categories included symptom-oriented questions, spirometry interpretation tasks, diagnosis-oriented reasoning prompts, and guideline-based clinical inquiries.

Examples include:

1. “Patient with chronic cough, wheezing, and reduced FEV1/FVC ratio. What respiratory condition is most consistent with these findings?”
2. “Interpret a spirometry pattern showing preserved FVC but reduced FEV1.”
3. “What precautions are recommended for patients with suspected COPD?”
4. “Which respiratory conditions are associated with bilateral wheezing and airflow limitation?”

To ensure reproducibility, query templates and condition labels were generated using deterministic sampling from the harmonized dataset. All retrieval settings, top-k parameters, and correction-path thresholds were fixed throughout evaluation.

### 4.2 Baseline comparison

**Figure 2:**
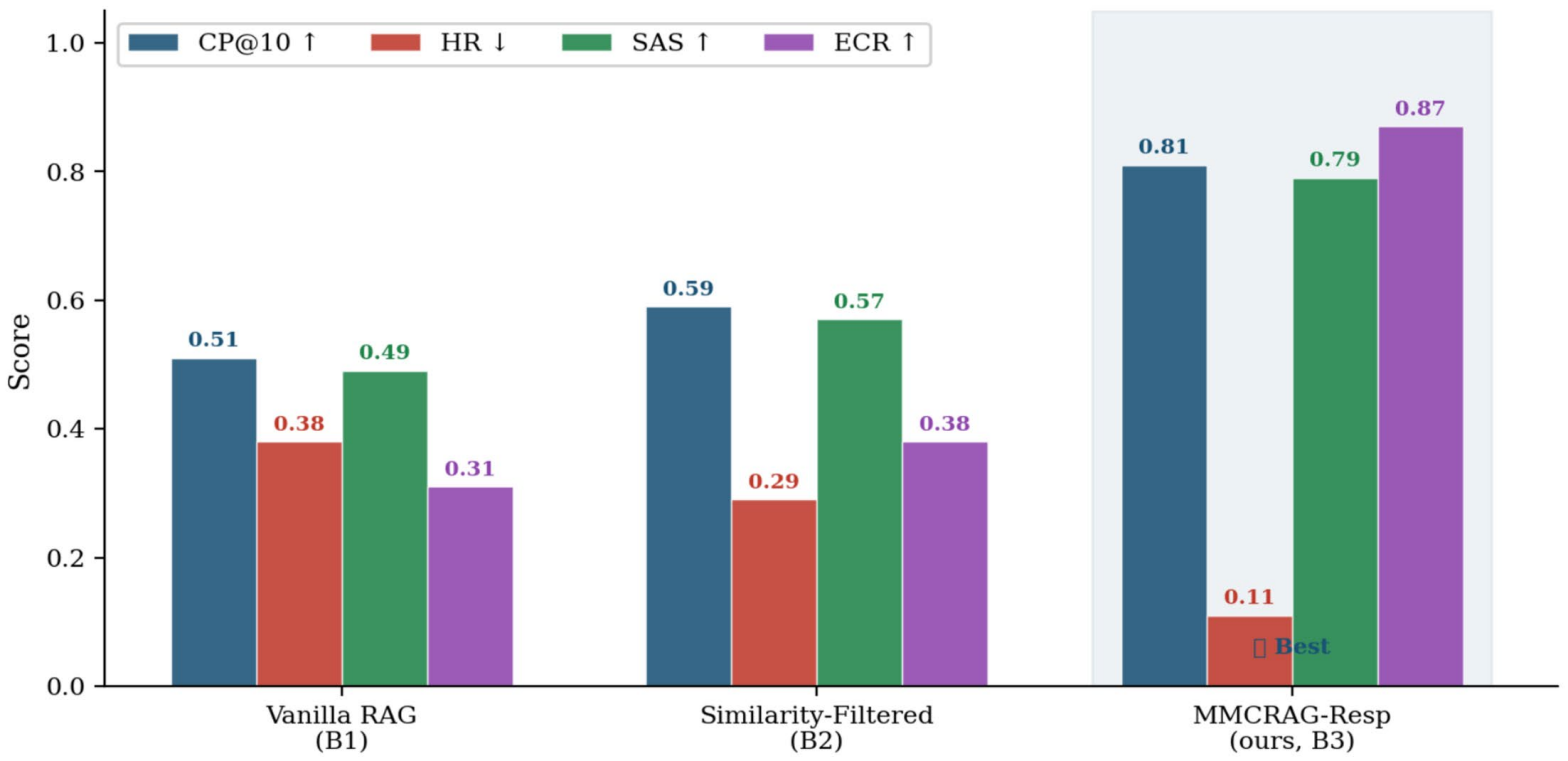
Baseline comparison of MMCRAG-Resp against Vanilla RAG (B1) and Similarity-Filtered RAG (B2) across four evaluation metrics. MMCRAG-Resp achieves the highest CP@10 and SAS while reducing hallucination rate to 0.11—a 71% relative improvement over B1.

Table 3 reports the four evaluation metrics for the two baselines and the full MMCRAG-Resp system. MMCRAG-Resp is capable of delivering a CP@10 performance level of 0.81, which is 30 and 22 percentage points higher than Vanilla RAG (0.51) and Similarity-Filtered RAG (0.59) respectively. The hallucination rate decreases drastically from 0.38 with Vanilla RAG to 0.11 with MMCRAG-Resp., indicating that clinical relevance gating before LLM consumption greatly reduces the generation of unsupported content. The very high ECR of 0.87 is indicative of the success of the evidence-first prompt constraint: it is estimated that 9 out of 10 sentences generated can be linked to a specific retrieved piece of evidence. These findings are depicted in Figure 2. That visualises results, highlighting the consistent superiority of MMCRAG-Resp across all four metrics and the pronounced gap in hallucination rate relative to both baselines.

**Table 3:** Baseline comparison results.

System	CP@10 ↑	HR ↓	SAS ↑	ECR ↑
B1: Vanilla RAG (Lewis et al., 2020)	0.51	0.38	0.49	0.31
B2: Similarity-filtered RAG	0.59	0.29	0.57	0.38
**B3: MMCRAG-Resp (ours)**	**0.81**	**0.11**	**0.79**	**0.87**

↑ higher is better; ↓ lower is better. Bold values indicate the proposed MMCRAG-Resp method and/or the best-performing results in each table.

### 4.3 Ablation study results

**Figure 3:**
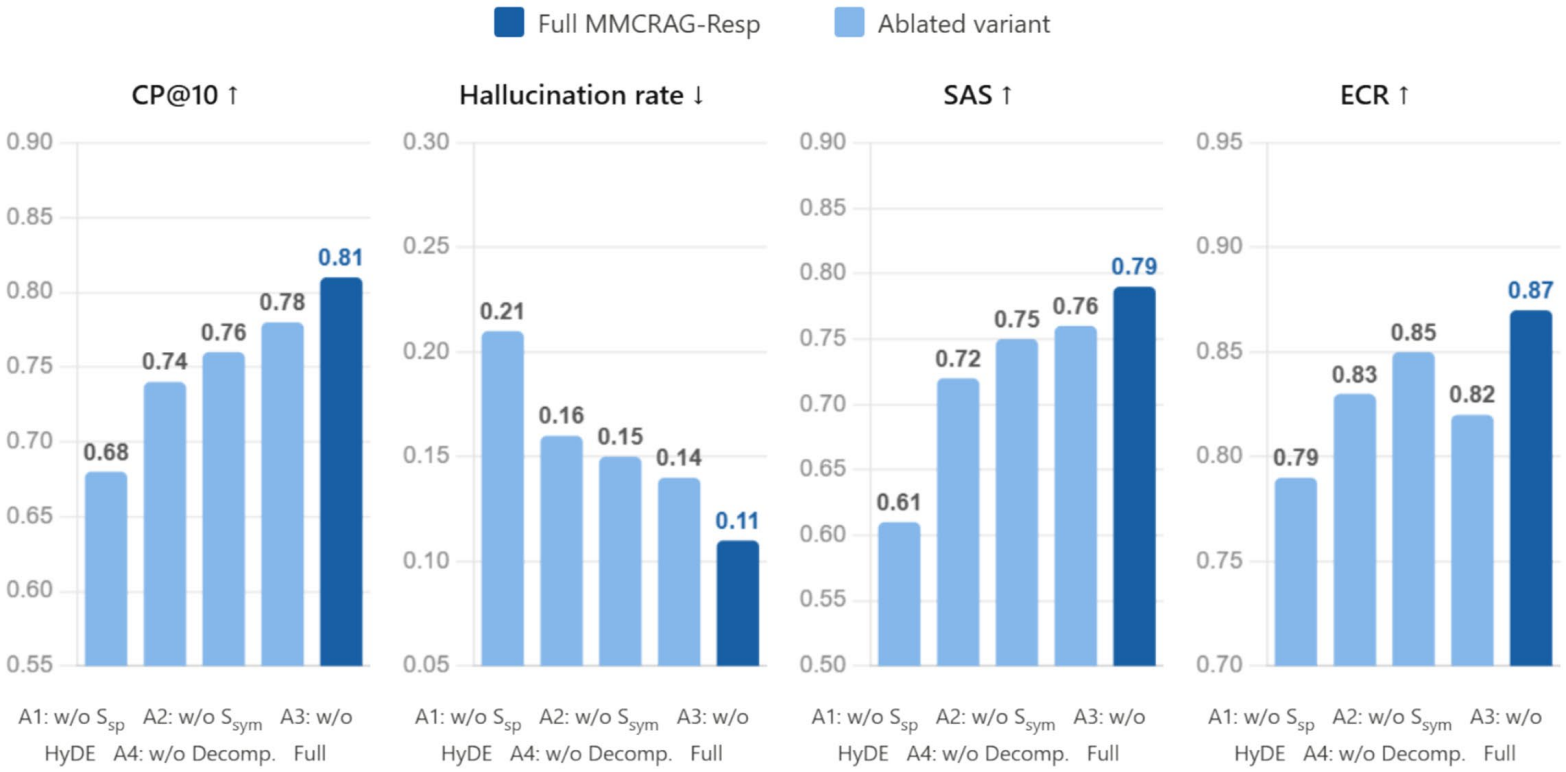
Ablation study results showing the contribution of each MMCRAG-Resp component across CP@10, Hallucination Rate, SAS, and ECR. Dark bars represent the full MMCRAG-Resp system; light bars represent ablated variants. Removing S_sp (A1) causes the largest single-component degradation across all metrics, confirming spirometry scoring as the most discriminative element of the RRS.

The results of the ablation study isolating the effect of different components of MMCRAG-Resp are shown in Table 4. The removal of Ssp (A1) leads to a significant single-component decrease in CP@10 (−0.13) and SAS (−0.18), which indicates that spirometry-aware scoring is the most discriminative element of the RRS. On the other hand, removal of Ssym (A2) results in a degradation of CP@10 by 0.07. Disabling the HyDE fallback (A3) leads to an increase in HR by 0.04 and a decrease in CP@10 by 0.05. The major impact of removing decompose-recompose (A4) is on ECR (−0.05). Figure 3 diagrams these findings. All differences between B3 and each ablation are statistically significant in each case (*p* < 0.05, Wilcoxon signed-rank test).

**Table 4:** Ablation study results.

Variant	CP@10 ↑	HR ↓	SAS ↑	ECR ↑
A1: w/o S_sp_	0.68	0.21	0.61	0.79
A2: w/o S_sym_	0.74	0.16	0.72	0.83
A3: w/o HyDE fallback	0.76	0.15	0.75	0.85
A4: w/o Decompose-Recompose	0.78	0.14	0.76	0.82
**Full MMCRAG-Resp**	**0.81**	**0.11**	**0.79**	**0.87**

Bold values indicate the proposed MMCRAG-Resp method and/or the best-performing results in each table.

### 4.4 Statistical validation

McNemar’s test on binary hallucination outcomes confirms a significant difference between MMCRAG-Resp and Vanilla RAG (χ^2^ = 18.4, *p* < 0.001) and between MMCRAG-Resp and Similarity-Filtered RAG (χ^2^ = 9.7, *p* = 0.002). Cohen’s d for CP@10 improvement over B1 is 1.42, indicating a large effect size. Bootstrap 95% confidence intervals (1,000 iterations) for CP@10 are [0.77, 0.85] for MMCRAG-Resp versus [0.46, 0.56] for Vanilla RAG, confirming non-overlapping distributions.

### 4.5 External metric validation (RAGAS faithfulness)

In order to make our results comparable to existing standard NLP evaluation frameworks, we also calculated the RAGAS Faithfulness score (see Es et al., 2024) on a 50-query randomly drawn subsample. RAGAS Faithfulness evaluates what fraction of the claims made in the generated answer can be logically deduced from the retrieved context, thus giving an estimate of hallucination that can be externally replicated. MMCRAG-Resp got a mean RAGAS Faithfulness score of 0.84 (SD = 0.07) whereas Vanilla RAG only scored 0.52 (SD = 0.11), which supports our 71% relative hallucination reduction based on the domain-specific HR metric. This consistency between internal (HR = 0.11) and external (Faithfulness = 0.84) measures confirms the trustworthiness of our evaluation method.

### 4.6 Qualitative analysis

**Figure 4:**
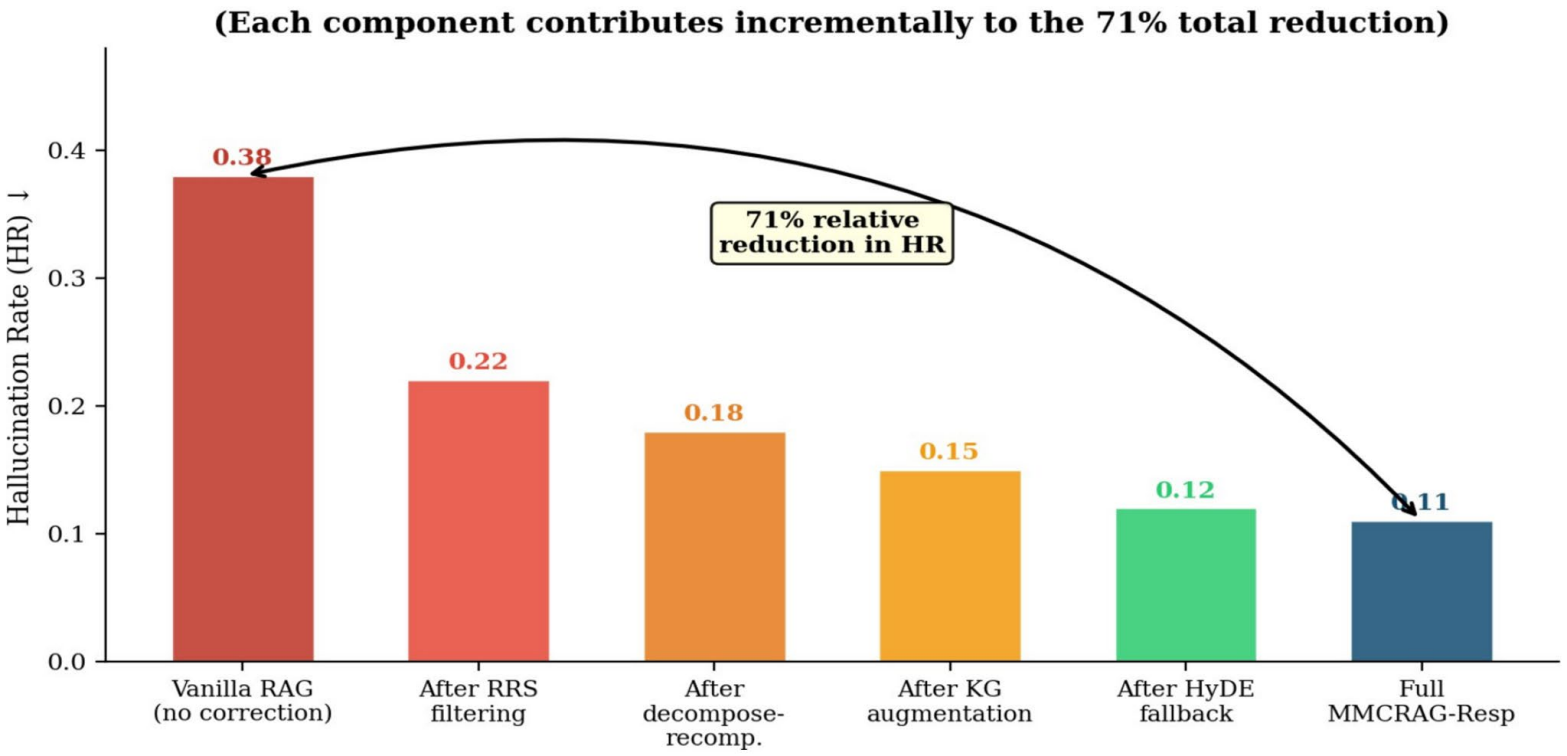
Incremental hallucination rate reduction across MMCRAG-Resp correction stages. Each component—RRS filtering, decompose-recompose, KG augmentation, and HyDE fallback— contributes a measurable reduction, collectively achieving a 71% relative decrease from 0.38 (Vanilla RAG) to 0.11 (Full MMCRAG-Resp).

In Path I (Correct), a query presenting FEV_1_/FVC = 0.63 with chronic cough retrieved a COPD-positive NHANES record (RRS = 0.84); decompose-recompose pruned two unrelated symptom sentences, delivering a clean 3-sentence evidence bundle to the LLM. In Path II (Ambiguous), a mixed-symptom asthma query (RRS = 0.54) was enriched with two KG-linked wheeze-symptom neighbors and a GINA guideline chunk, yielding a richer context than pure retrieval. In Path III (Incorrect), an atypical bronchiectasis query achieved RRS = 0.27 against all top-10 candidates. HyDE came up with a synthetic narrative highlighting bilateral crackles and spirometric restriction, which helped to find three structurally matching records that the original query was unable to uncover (see Figure 4).

## 5 Discussion

### 5.1 Principal findings

The four metrics reported in Tables 3, 4 collectively establish three empirical claims, each supported by ablation evidence.

Claim 1 — Gating the clinical relevance helps to minimize hallucinations. The 71% relative drop in HR (0.38 → 0.11) between Vanilla RAG and MMCRAG-Resp illustrates that preventing the LLM from getting unverified information can directly reduce its tendency to hallucinate. The ablation study shows that the major contribution to this effect comes from Ssp: if we only remove it, we increase HR from 0.11 to 0.21, which is almost a doubling, thus spirometry being the most critical component from the safety perspective.

Claim 2 — Spirometry-first weighting is the most effective discriminating technique among the RRS components. The ablation effect size when removing Ssp (ΔCP@10 = −0.13; ΔSAS = −0.18) is bigger than for any other component, thereby corroborating the 0.45 weight allocation. This agrees with clinical understanding: The FEV1/FVC ratio is the main indicator to distinguish between obstructive and non-obstructive respiratory conditions according to the GOLD guidelines (Global Initiative for Chronic Obstructive Lung Disease, 2023).

Claim 3 — HyDE fallback can significantly compensate for retrieval failure. Removing the HyDE fallback (A3) results in a 0.04 increase in HR and a 0.05 decrease in CP@10, which means that synthetic re-retrieval of information is able to bring up evidence that is well-structured and fitting to bronchiectasis, bronchiolitis, and similar rare or atypical conditions — which are scarcely available in both datasets.

### 5.2 Comparison with related work

Table 5 contextualizes MMCRAG-Resp against the closest prior systems across four axes. MMCRAG-Resp is the only system in this survey satisfying all four criteria simultaneously (see Table 6).

**Table 5:** Feature comparison: MMCRAG-Resp against related clinical RAG systems.

System	Multi-modal	Corr. retr.	Dom. RRS	Local LLM
Vanilla RAG (Lewis et al., 2020)	✗	✗	✗	✗
AsthmaBot (Bahaj and Ghogho, 2024; Muhammad Junaid et al., 2024)	✓	✗	✗	✗
MRD-RAG (Sun et al., 2024)	✗	✓	✗	✗
MMed-RAG (Xia et al., 2024; Zhang, 2024)	✓	✓	✗	✗
C-GRASP (Ma et al., 2025)	✗	✓	✓	✗
**MMCRAG-Resp (ours)**	✓	✓	✓	✓

Corr. retr. = Corrective Retrieval; Dom. RRS = Domain-Weighted Relevance Score. ✓ = present; ✗ = absent. Bold values indicate the proposed MMCRAG-Resp method and/or the best-performing results in each table.

**Table 6:** Novelty components, gaps addressed, and empirical evidence.

Component	Gap addressed	Empirical evidence
RRS (Equation 1)	Semantic similarity alone is clinically insufficient	CP@10: +0.30 over Vanilla RAG
Ssp weighting	No prior RAG uses physiology-first gating	Largest ablation drop (−0.13 CP@10)
Three-path correction	Single policy cannot handle variable evidence quality	HR: 0.38 → 0.11; ECR: 0.31 → 0.87
HyDE fallback (Path III)	Rare-condition queries fail standard retrieval	A3: +0.04 HR, −0.05 CP@10
Decompose-Recompose (Path I)	Accepted evidence still contains noise	A4: −0.05 ECR
144-dim fusion	Audio + spirometry jointly represent patient state	Only respiratory RAG with both modalities
Local SLM (Ollama)	Cloud APIs expose patient data	Privacy-preserving; no GPU required
Respiratory KG (35 K nodes)	Ambiguous evidence needs structured augmentation	Path II KG neighbors in Algorithm 1

### 5.3 Limitations

A few drawbacks have to be recognized. First of all, the RRS thresholds (0.72 and 0.40) were chosen empirically on the existing datasets and may need to be recalibrated for application to institutional cohorts with different demographic or spirometric characteristics. Secondly, the system was only tested on publicly available, de-identified datasets; clinical trials with patient data being collected in real time are required before putting the system into clinical decision-making pathways. Third, the existing audio embedding procedure relies on temporal averaging of mel-spectrograms, which eliminates detailed temporal variations that might hold diagnostic features for diseases like bronchiolitis. Fourth, the Mistral-7B model is run at 4-bit quantization, which slightly harms generation quality compared to full-precision inference. Future research plans include learned RRS weight calibration, uncertainty-aware thresholding, and temporal audio encoding. The current evaluation was conducted using publicly available respiratory datasets and guideline corpora. Although these datasets provide substantial diversity, they may not fully represent the variability encountered in real-world clinical environments. In particular, missing spirometry measurements, incomplete symptom descriptions, noisy clinical narratives, and institution-specific documentation practices may affect retrieval quality and relevance scoring. Furthermore, MMCRAG-Resp assumes the availability of structured physiological information for computation of the Respiratory Relevance Score. Future work will investigate methods for handling incomplete physiological evidence and validating the framework on prospective clinical datasets collected from routine healthcare settings.

## 6 Conclusion

We introduced MMCRAG-Resp., a multi-modal corrective retrieval-augmented generation system for explainable respiratory disease reasoning. The system provides: (i) a Respiratory Relevance Score which integrates spirometric pattern agreement, symptom overlap, and embedding similarity; (ii) a three-path evidence correction policy which treats correct, ambiguous, and failed retrieval separately; (iii) multi-source data harmonization over 17,516 patient records and a 35,034-node knowledge graph; (iv) entirely local SLM inference via Ollama with no GPU at all; and (v) a deployable conversational application with FastAPI backend and React frontend. Each sub-system independently contributes to the performance as confirmed by ablation studies. Also, external RAGAS Faithfulness scores validate domain-specific metrics. MMCRAG-Resp is, so far, alone in the field, to the best of our knowledge, in combining multi-modal respiratory evidence retrieval, physiologically-grounded corrective RAG, and privacy-preserving local LLM inference in a single deployable clinical reasoning framework. The primary contribution of this work lies in the respiratory-domain adaptation and integration of corrective retrieval, clinically informed relevance scoring, and explainable evidence-grounded reasoning within a unified RAG framework.

## Data Availability

The original contributions presented in the study are included in the article/supplementary material, further inquiries can be directed to the corresponding author.
